# A Review of Machine Learning Approaches to Eye Disease Detection: Current Trends, Challenges and Future Directions

**DOI:** 10.3390/diagnostics16213546

**Published:** 2026-10-28

**Authors:** Matthias Omotayo Oladele, Oyeniyi Akeem Alimi, Oludayo O. Olugbara

**Affiliations:** 1 Department of Information Technology, Durban University of Technology, Durban 4001, South Africa; 2 Department of Information Systems, Durban University of Technology, Durban 4001, South Africa; oyeniyia@dut.ac.za; 3 MICT SETA 4IR Center of Excellence, Durban University of Technology, Durban 4001, South Africa; oludayoo@dut.ac.za

**Keywords:** deep learning, ensemble learning, explainability, eye disease detection, machine learning, vision transformer

## Abstract

**Background/Objectives**: Vision impairment and blindness remain a major global health challenge, with glaucoma, diabetic retinopathy, age-related macular degeneration, cataract, corneal disease, and retinopathy of prematurity representing important causes of preventable or treatable vision loss when detected early. This clinical need has accelerated research on machine learning approaches for automatic detection of eye diseases, grading, and segmentation. However, existing reviews often focus on a single disease or method. They also give limited attention to their suitability and applicability for reliable clinical deployments. This review synthesizes traditional machine learning, deep learning, vision transformers, ensemble and hybrid models, segmentation methods, and explainable AI approaches for eye disease detection. It further examines task formulation, dataset characteristics, evaluation practices, and deployment constraints, with emphasis on clinical readiness rather than benchmark performance alone. **Methods**: A structured narrative review was conducted using recently published articles from reputable databases and repositories, including IEEE Xplore, PubMed/MEDLINE, ScienceDirect, Scopus and arXiv. The review critically analyzes the progression from traditional machine learning to advanced models, including deep learning, vision transformers, ensemble models and explainable artificial intelligence. Studies were critically analyzed according to task formulations, dataset characteristics, segmentation approaches, evaluation metrics, explainability, and deployment constraints. **Results:** The synthesis shows that class imbalance, limited demographic and geographic diversity in available datasets, weak external validation, and scarce prospective evidence continue to separate benchmark performance from clinical readiness. Explainability is also often evaluated through visual plausibility rather than validated faithfulness. Currently, data-driven diabetic retinopathy screening is closest to clinical readiness, particularly for distinguishing referable from non-referable cases, whereas multi-label detection, rare disease detection, and cross-device deployment remain at the research stage. **Conclusions**: Progress towards clinical adoption requires stronger alignment between experimental evaluation and real-world deployment. This includes external validation and the development of equitable, interpretable, and resource-aware systems.

## 1. Introduction

Eye disease is a major global health challenge. The World Health Organization estimates that at least 2.2 billion people worldwide live with vision impairment, and that at least one billion of these cases are preventable or remain unaddressed [1]. Much of this impairment is attributable to a few chronic eye diseases, namely diabetic retinopathy (DR), glaucoma, age-related macular degeneration (AMD), cataract, corneal opacities, and retinopathy of prematurity (ROP). DR affected approximately 103 million adults in 2020, a figure projected to reach 160 million by 2045 [2]. Glaucoma, globally recognized as the leading cause of irreversible blindness, affected an estimated 76 million people in 2020 and is expected to rise to 112 million by 2040 [3]. AMD, cataract, corneal opacities, and ROP together account for millions of additional cases of avoidable vision loss [4]. These statistics and their associated effects fall disproportionately on low- and middle-income countries, where limited specialist infrastructure and restricted screening coverage often delay efficient diagnosis, mitigation and early treatment. Treatment is typically less effective and quite expensive when diagnosis is delayed [5]. Conventional screening methods such as ophthalmoscopy and slit-lamp biomicroscopy require trained clinicians, examination time, and specialized infrastructure, which limits scalability and reproducibility [6]. This reliance on specialist assessment restricts screening coverage where specialists are scarce. For example, sub-Saharan Africa has approximately 2.5 ophthalmologists per million people [7]. This is quite disproportionate to the WHO recommended target of at least 4 ophthalmologists per million people. These specialist and infrastructure constraints, coupled with the rising burden of vision-threatening diseases, create a need for scalable, cost-effective diagnostic tools that can extend screening capacity and reduce reliance on specialist assessment.

Machine learning (ML) models present a viable non-invasive approach for automatic detection, grading, and classification of eye diseases using retinal images and clinical data. Early approaches applied classical ML methods such as support vector machines (SVMs), decision trees (DTs), and logistic regression (LR) to hand-engineered image features [8,9,10]. Recent works rely on advanced ML models, predominantly deep learning models, including convolutional neural networks (CNNs), vision transformers (ViTs), and ensemble methods for eye disease analysis [11,12,13,14]. Compared with conventional models, these advanced ML methods can process voluminous retina images more quickly and reliably [15,16]. A demonstration of this capability was reported in the study by Gulshan et al. [17], whereby a modelled deep learning algorithm for detecting referable DR matched the performance of United States board-certified ophthalmologists. Subsequent research extended the approach to other imaging modalities, with Kermany et al. [18] applying a CNN-based transfer learning model for optical coherence tomography (OCT) analysis. From the experimentation, classification was successfully done across choroidal neovascularization (CNV), diabetic macular edema (DME), drusen, and normal categories. More recent studies have progressed towards attention-based architectures, with Swin Transformer-based models outperforming CNN baselines on multi-disease fundus classification [14]. Similarly, in [19], Transformer models report high accuracy on curated benchmarks, around 99% on OCT2017. However, these results come from single-source data without external validation across multiple datasets. 

Prior reviews of AI-based eye disease analysis [20,21,22] have provided valuable insights. Ting et al. [20] reviewed AI and deep learning with an emphasis on DR. A systematic review of machine learning on tabular data for glaucoma detection was reported by Shahriari et al. [21]. A review of deep learning for automated detection across glaucoma, DR, and macular degeneration was presented by Dash et al. [22]. While these reviews largely organize the literature around what models can detect and how they score on benchmarks, few critically analyze the methodologies comprehensively or evaluate the conditions under which the proposed models’ performances would transfer to clinical use. This leaves a gap between cataloguing methodological progress and assessing clinical readiness. This study aims to build on these reviews with an updated review of recent studies, and, more importantly, it addresses the identified gaps by organizing the literature around clinical readiness rather than benchmark performance alone. It critically synthesizes ML and deep learning approaches for eye disease detection across methods, diseases, task formulations, and datasets. It further evaluates each approach against the structural conditions that determine whether reported results transfer to practice, including external validation, class imbalance, demographic and geographic diversity, deployment feasibility, and the validity of model explanations. Additionally, the study highlights key limitations for current research trends in this field and directions for future studies. 

This study uses a structured narrative review rather than a PRISMA-guided systematic review. Focusing on recently published articles (mainly over the past five years) in prominent scientific databases and repositories including IEEE Xplore, PubMed/MEDLINE, ScienceDirect, Scopus and arXiv, this review critically evaluates the strengths and limitations of current ML models’ methodological approaches, including the deployed datasets, data processing and segmentation methods used for eye disease detection.

The key contributions of this review study are as follows:

- Review of recent ML approaches for detecting and classifying major eye diseases;
- Synthesis of traditional ML, deep learning and CNN architectures, ViT and attention mechanisms, ensemble and hybrid methods, and explainable AI techniques;
- Analysis of binary, multi-class, and multi-label classification types;
- Critical examination of public datasets, benchmarks, and evaluation frameworks, with attention to their limitations in real-world and low-resource settings;
- Identification of challenges, limitations, and research gaps, with directions for clinically deployable eye disease detection using AI.

The remainder is organized as follows. Section 2 outlines the review methodology. Section 3 examines the major eye diseases and their clinical significance. Section 4 comprehensively analyzes ML approaches, while Section 5 critically analyzes current datasets that are typically deployed for classification and segmentation tasks. Section 6 reviews classification tasks. Section 7 reviews segmentation approaches, while Section 8 discusses the explainability and interpretability of models’ results, and Section 9 identifies the research gaps and future directions. Section 10 concludes the study.

## 2. Materials and Methods

The study adopts a structured narrative review to examine recent developments in machine learning for eye disease detection, with particular emphasis on the gap between reported benchmark performance and clinical readiness. The literature search covered IEEE Xplore, PubMed/MEDLINE, ScienceDirect, Scopus, and arXiv. Search strings combined eye disease terms with method-related terms. This includes “machine learning”, “deep learning”, “eye disease”, “diabetic retinopathy”, “glaucoma”, “age-related macular degeneration”, “cataract”, “retinal fundus”, “optical coherence tomography”, “vision transformer”, “explainable artificial intelligence”, “segmentation”, and “ophthalmology”. These terms were combined using Boolean operators in the general form as (“machine learning” OR “deep learning” OR “artificial intelligence” OR “convolutional neural network” OR “vision transformer” OR “transfer learning” OR “ensemble learning” OR “foundation model” OR “self-supervised learning” OR “federated learning” OR “explainable artificial intelligence”) AND (“eye disease” OR “retinal disease” OR “diabetic retinopathy” OR “diabetic macular edema” OR “glaucoma” OR “age-related macular degeneration” OR “cataract” OR “retinopathy of prematurity” OR “corneal disease” OR “retinal fundus” OR “fundus photography” OR “optical coherence tomography”) AND (“detection” OR “classification” OR “grading” OR “screening” OR “diagnosis” OR “segmentation”), adapted to the syntax of each database. The search included articles from 2020 to 2026. This window was chosen deliberately to capture the rapid methodological shift toward deep learning, vision transformers, and foundation models that has defined the field since 2020. Rather than providing an exhaustive historical account, a small number of earlier works were retained only where they introduced foundational datasets, architectures, or clinical concepts that remain in active use.

Studies were included on three grounds. The first was the application of ML or deep learning to eye disease detection, grading, classification, segmentation, or clinically relevant decision support. The second was the introduction or characterization of an eye imaging dataset. The third was a substantial methodological or translational contribution to the field. A study met this criterion if it introduced a new architecture and/or training approach, dataset, evaluation framework, deployment insight, etc., which is/are directly relevant to the clinical-readiness focus of this review. Excluded sources were those without verifiable bibliographic records, papers unrelated to eye disease, and studies lacking sufficient methodological detail. The synthesis grouped studies by disease target, imaging modality, model family, classification type, segmentation task, dataset type, and translational barrier. Performance values were interpreted according to dataset size, validation setting, class balance, external testing, explainability, and deployment feasibility so that headline accuracy is not treated as equivalent to clinical readiness. Consistent with a narrative rather than systematic review, records were not formally enumerated per database, and no PRISMA flow diagram or risk-of-bias assessment was produced. The selection instead prioritized breadth, methodological relevance, and clinical significance. This is acknowledged as a limitation in Section 9.5. Table 1 summarizes the sources, search terms, and selection approach.

## 3. Overview of Common Eye Diseases

This section examines the major eye diseases studied in AI-based detection, including DR, glaucoma, AMD, cataract, and ROP. Although these conditions differ in their clinical features and imaging requirements, each presents specific challenges for automated analysis. These differences therefore influence how detection tasks are formulated, which imaging data are most suitable, and how model performance should be interpreted. It is important to note that each disease carries a different reference standard, clinical pathway, and deployment requirement. Claims of clinical readiness are therefore necessarily disease- and task-specific and cannot be generalized across all eye conditions. For example, autonomous screening for referable diabetic retinopathy is different from glaucoma risk stratification or cataract grading.

### 3.1. Diabetic Retinopathy

Diabetic retinopathy (DR) is a microvascular complication of diabetes that damages the retinal vasculature. If untreated, it progresses from mild non-proliferative DR (NPDR) to proliferative disease, which can lead to vision loss [23]. This progression is formalized in the International Clinical Diabetic Retinopathy (ICDR) scale, which grades DR from no apparent retinopathy through mild, moderate, and severe NPDR to proliferative DR [24]. The clinical signs escalate in step across these stages. Mild NPDR is characterized by isolated microaneurysms. Moderate and severe NPDR add hemorrhages, hard exudates, cotton wool spots, and venous beading. Proliferative disease is marked by neovascularization and pre-retinal or vitreous hemorrhage [25]. Each of these stage-defining lesions is a visible image feature that a model can be trained to detect directly. This makes DR highly tractable for automated analysis. The same lesions, however, define the difficulty of the task. The earliest and most screening-relevant grade is signaled by microaneurysms. These are small, low in contrast, and few, and therefore difficult to detect reliably. This difficulty is compounded by the fact that DR is asymptomatic until it is advanced [2]. Thus, the clinical value of detection falls precisely on the mild and moderate grades that a patient cannot feel, and a model struggles to see. It is this concentration of value at the hardest-to-detect stages that justifies automated screening of diabetic populations.

In clinical practice, the screening target is not DR in general but specific, actionable endpoints. These are referable DR (moderate NPDR or worse, or any DME), vision-threatening DR (severe NPDR and proliferative DR), and center-involving DME, which carries the highest risk of acuity loss. This distinction matters for automated detection because the clinically decisive output is a threshold decision at the referable level rather than a fine-grained severity grade. This framing is reflected in the first autonomous AI systems cleared for DR screening. IDx-DR [26] and EyeArt [27] output a binary referral decision, referable versus non-referable, at a fixed operating point rather than a full five-level severity grade. This design aligns their outputs with the clinically actionable endpoint. Much of the research literature, by contrast, optimizes multi-class grading accuracy on curated benchmarks without defining an operating threshold. This can inflate apparent performance, and it leaves the clinically decisive quantity, sensitivity, at a fixed specificity under-reported. The same five-level severity structure also shapes how DR is modeled, since the task is often posed as a multi-class grading rather than binary detection. These attributes further explain why class imbalance is severe, as referable and proliferative cases are rare relative to no-DR images in any screening population.

### 3.2. Glaucoma

Glaucoma is a group of progressive optic neuropathies characterized by optic nerve head damage, retinal ganglion cell loss, and visual-field defects [28]. It is a leading cause of irreversible blindness yet may remain asymptomatic until substantial damage has occurred [29]. The clinical objective is therefore to detect structural change before substantial functional loss occurs. Early detection is particularly important, since timely intervention can reduce the risk of further visual-field deterioration [30]. However, this remains challenging because glaucomatous changes can be subtle and may overlap with normal variation in optic nerve head morphology [31]. Unlike diabetic retinopathy, where automated analysis commonly focuses on discrete retinal lesions, glaucoma detection relies mainly on morphological changes in the optic nerve head [32]. Fundus-based methods assess these changes through measures such as the cup-to-disc ratio (CDR) [33], while optical coherence tomography provides measurements of retinal nerve fiber layer thickness [34]. Although CDR is widely used, its interpretation is influenced by physiological variation in optic disc size and morphology. A key distinction is that fundus-based models typically identify glaucoma suspects or signs of optic-disc cupping rather than diagnosing glaucoma itself. Definitive diagnosis instead integrates intraocular pressure, standard automated perimetry, OCT, and clinical examination. Automated fundus analysis is therefore best positioned as a triage or case-finding step rather than a standalone diagnostic test. 

This dependence on optic nerve head geometry shapes the computational task. Glaucoma is commonly framed as both image-level classification and segmentation [35]. Image-level classification separates glaucomatous from non-glaucomatous eyes, while segmentation delineates the optic disc and cup to estimate the CDR. Segmentation therefore remains important in morphology-based glaucoma assessment because it converts subtle structural changes into measurable features such as CDR, although its value depends on accurate delineation of the optic disc and cup [31]. However, it is noteworthy to highlight that segmentation is not a prerequisite for glaucoma classification, as models can learn discriminative features directly from fundus images.

### 3.3. Age-Related Macular Degeneration and Macular Disorders

As the name implies, AMD is a major cause of severe vision loss among older adults [36]. It primarily affects photoreceptors, retinal pigment epithelium, Bruch membrane, and choriocapillaris [37]. AMD damages the macula and impairs reading, face recognition, and driving [38]. It progresses from early and intermediate disease, marked by small-to-large drusen and pigmentary change, to advanced disease, which takes two forms, dry and wet AMD. Dry AMD is characterized by geographic atrophy involving the fovea, while wet AMD involves choroidal neovascularization with exudation, hemorrhage, or scarring [36]. Wet AMD is less common but causes more rapid and severe decline, since abnormal choroidal vessels leak fluid or blood into the macula [39]. 

This staged pathology shapes the computational task. Binary classification may overlook clinically important differences between disease stages, making severity classification and biomarker detection more informative. The imaging modality also determines what can be detected. Fundus imaging reveals drusen, geographic atrophy, and hemorrhage, whereas OCT provides cross-sectional detail on established biomarkers such as drusen volume, intraretinal and subretinal fluid, pigment epithelial detachment, retinal-layer disruption, and hyper-reflective foci [40]. These biomarkers are central to staging and to distinguishing geographic atrophy in advanced dry AMD from neovascular activity in wet AMD. This distinction is especially important in wet AMD, where OCT biomarkers are used to assess disease activity and treatment response. Automated AMD systems extend beyond diagnosis to biomarker localization, severity assessment, and treatment monitoring.

### 3.4. Cataract

Cataract is a leading global cause of blindness, affecting approximately 65 million people with vision impairment or blindness [1,41]. It occurs when the crystalline lens loses transparency through progressive opacification, as crystallin misfolding and aggregation generate insoluble, light-scattering deposits [42]. This causes blurred vision, reduced contrast sensitivity, glare, and progressive loss of functional vision, with age-related cataract the most common subtype [43]. Cataract affects the lens rather than the retina, so assessment relies on anterior-segment imaging such as slit-lamp biomicroscopy, retro-illumination, and standardized lens-opacity grading, most commonly the Lens Opacities Classification System III (LOCS III) [44,45]. Definitive treatment is surgical extraction with intraocular lens implantation, while pharmacological therapy is limited [46]. Importantly, the decision to operate depends not on opacity grade alone but on visual acuity, the patient’s symptoms and functional needs, and biometry for intraocular lens selection. An automated opacity grade is only one input to a broader surgical decision. Detection using AI therefore targets lens opacity, severity grading, subtype classification, and preoperative decision support rather than vascular or macular pathology.

Despite its importance, cataract remains underrepresented in AI research relative to DR, glaucoma, and AMD [47]. This reflects the scarcity of large annotated cataract datasets and a reliance on anterior-segment imaging that differs from the fundus repositories dominating multi-disease detection. Among public benchmarks, ODIR-5K is one of the few multi-disease datasets that include cataract as a target class, though its fundus-based design differs from the anterior-segment imaging used for direct cataract assessment.

### 3.5. Corneal Diseases and Retinopathy of Prematurity

Corneal diseases and ROP broaden the scope of AI-based eye disease detection beyond adult retinal and lens disorders. Corneal diseases such as keratoconus, corneal opacities, and infectious keratitis cause extensive monocular blindness, especially in low-income settings where trauma and infection are more frequent [48]. Their assessment relies on dedicated anterior-segment imaging, including corneal topography, Scheimpflug tomography, slit-lamp photography, and anterior-segment OCT, rather than the fundus imaging used for retinal disease. CNN-based models have targeted keratoconus detection and corneal-ulcer classification, but the absence of large public corneal datasets limits development and external validation [49]. ROP is an important cause of childhood blindness in middle-income countries [50], and CNN models have achieved expert-comparable performance for plus-disease detection [51]. Clinically, ROP screening is driven by gestational age and birth weight to identify at-risk neonates. Screening then relies on serial examinations to track disease progression, so a single image classified in isolation does not reflect how ROP is managed in practice [52]. Multi-center ROP datasets from Sub-Saharan African and South Asian neonatal units also remain critically limited. Corneal disease and ROP therefore illustrate a recurring pattern in which the availability of data, rather than the clinical importance of a condition, determines where automated detection develops.

## 4. Machine Learning Approaches to Eye Disease Detection

**Figure 1:**
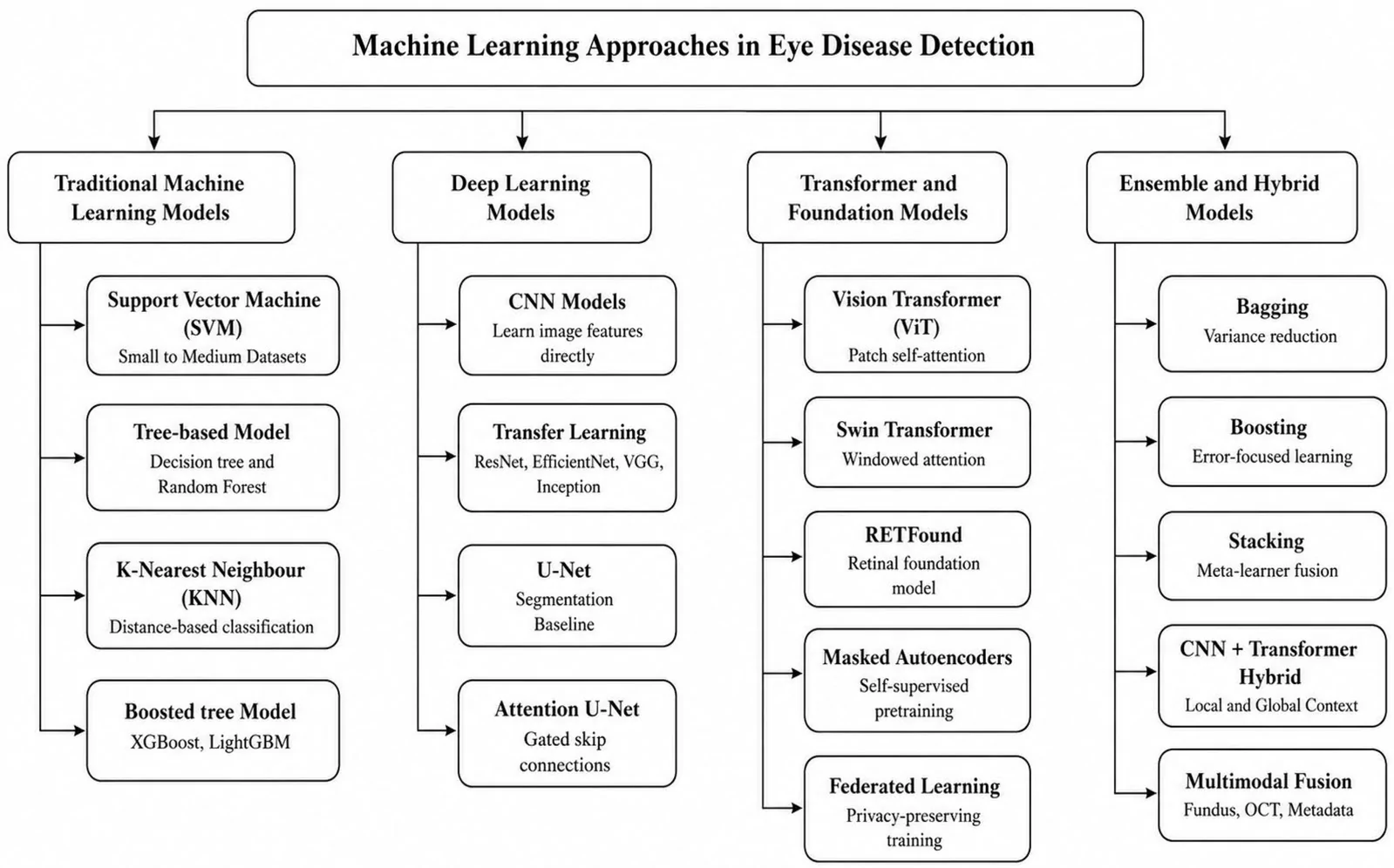
Overview of machine learning approaches used in the detection of eye diseases [8,57,58,59]. Abbreviations: XGBoost—Extreme Gradient Boosting; LightGBM—Light Gradient Boosting Machine; CNN—Convolutional Neural Network; ResNet—Residual Network; EfficientNet—Efficient Network; VGG—Visual Geometry Group; U-Net—U-shaped Convolutional Neural Network; RETFound—Retinal Foundation Model; OCT—Optical Coherence Tomography.

Eye images contain complex structural patterns that support screening, grading, segmentation, and decision support [53]. Fundus images and OCT scans capture retinal lesions, macular changes, and optic nerve morphology [54,55], while slit-lamp bio-microscopy and anterior-segment photography capture lens opacity [56]. Computational methods have evolved from feature-engineered classifiers to advanced ML models, including deep learning, transformer-based, and hybrid models. As shown in Figure 1, this section groups these approaches into four families: traditional ML, deep learning, transformer and foundation models, and ensemble and hybrid systems. Although they differ in architecture and learning strategy, they converge on three clinical functions: image-level classification, pixel-level segmentation, and integrated decision support that combines prediction with explainability, calibration, uncertainty estimation, and clinical metadata. This provides the basis for comparing performance, interpretability, scalability, and translational readiness. These families also trade accuracy against computational cost, since deeper models raise computational and deployment burden, creating a persistent tension between algorithmic sophistication and clinical scalability.

### 4.1. Traditional Machine Learning Models

Traditional ML provided the earliest computational approach to eye disease detection and remains valuable for lightweight, interpretable baselines where data, annotation, or compute are limited [60]. Supervised methods such as SVMs, DTs, multilayer perceptron neural networks, and random forests learn predictive mappings from labeled data [61], while unsupervised methods such as k-means and principal component analysis (PCA) identify latent structure in unlabeled data [62]. Reliance on manually extracted features limits scalability and makes performance highly sensitive to imaging device, acquisition method, and population [63].

Early feature extraction relied on signal and image processing to encode lesion texture, vessel morphology, and optic-disc structure [64]. This concern reveals the core limitation of traditional ML. Time-domain methods such as empirical mode decomposition capture pixel-level intensity variation for fine-grained analysis. Parashar and Agrawal [65] combined image-based EMD with PCA for multi-stage glaucoma classification. Frequency-domain methods such as the Fourier transform capture global texture but lose local spatial information, restricting representation of focal lesions [66]. This limitation motivated wavelet-based time-frequency methods, which preserve both local lesion structure and multi-scale texture [67], and which have supported multi-stage glaucoma classification [68] and multi-disease screening across DR, cataract, and glaucoma [69]. It is worth mentioning that in traditional ML, feature design must be tailored to each disease. Thus, a representation tuned for glaucoma rarely transfers. Also, performance depends on extensive preprocessing and manual parameter selection that degrades when lesion appearance shifts across imaging devices or demographic groups.

SVMs have been widely used in traditional ophthalmic ML-based modelling, particularly with handcrafted features and nonlinear kernels [8]. Tree-based models, boosting models such as XGBoost and LightGBM, and distance-based KNN have supported DR grading, glaucoma screening, and cataract classification [70]. Overall, considering that conventional ML-based approaches depend heavily on manual feature extraction, which results in poor generalization across datasets, they have contributed significantly to the shift toward deep-learning methods.

### 4.2. Deep Learning Models

Deep learning moved the field from handcrafted features towards end-to-end representation learning from raw images [71]. For example, in CNNs, hierarchical features are learned directly through successive convolution, activation, pooling, and normalization [72]. Thus, heterogeneous retinal patterns that manual feature extraction often misses will be effectively captured [73]. Typically, learning such representations from scratch demands large, labeled datasets, which are rare in ophthalmology. Transfer learning addresses this issue by fine-tuning ImageNet-pretrained models such as VGG16, ResNet50, InceptionV3, and EfficientNet on comparatively small ophthalmic datasets [71]. This approach produced the field’s earliest demonstrations of clinical feasibility. Gulshan et al. [17] reported ophthalmologist-level DR screening, and Kermany et al. [18] classified OCT scans across CNV, DME, drusen, and normal categories at 96.6% accuracy. These results established that deep networks could match expert performance on curated benchmarks. However, they may not hold in deployment, as both studies were validated on data from a small number of centers and imaging devices rather than across the populations and imaging conditions of routine screening. The same limitation applies to the pixel-level task, where U-Net is a dominant framework due to its encoder–decoder structure and skip connections that preserve spatial detail for optic-disc, cup, vessel, and lesion delineation [74]. Techniques such as augmentation, dropout, and label smoothing mitigate small samples and class imbalance [75]. However, they adjust for limited data rather than remove the underlying dependence on it.

### 4.3. Transformers and Foundation Models

ViTs process images as patch sequences. They typically use multi-head self-attention to model relationships among distant retinal regions [76]. This attribute specifically suits eye disease detection where relevant features span the macula, optic nerve head, and peripheral vasculature. Li et al. [19] applied a Swin Transformer V2 to OCT-based multi-fundus classification, reporting 99.9% accuracy on OCT2017 and 99.5% on OCT-C8. In a similar study, Rahman et al. [14] trained a Swin transformer on 16,242 Bangladeshi fundus images, reporting 91.00% accuracy and an F1-score of 0.9088 across ten conditions, outperforming VGG19, ResNet50, and SVM. These results, however, rely on curated, single-source datasets tested without external validation. Their strong benchmark performance therefore shows effective discrimination within the tested datasets, but they do not establish how well these models would generalize across different imaging devices and populations, or routine clinical settings.

As viable alternatives, foundation models extend this approach by pretraining on large unlabeled retinal datasets before fine-tuning. RETFound typically learns general retinal representations through self-supervised masked autoencoding and adapts to multiple downstream diseases with limited labels [58]. Federated learning trains shared models across institutions without exchanging patient images [77]. Their advantages are broader generalization and lower annotation cost. However, they tend to add coordination and communication overhead that low-resource sites may not sustain. Attention has also been incorporated into CNNs through squeeze-and-excitation blocks [78] and CBAM [79], which can improve DR grading, glaucoma screening, and AMD staging. They do not resolve the main translational barriers, since models often degrade on new imaging devices, populations, or disease distributions [80] and remain difficult to audit [81]. Reported advantages of transformer and foundation models over CNNs may therefore reflect dataset size, preprocessing, disease prevalence, or validation design rather than architecture. Therefore, any claim of clinical superiority requires external validation, calibration analysis, uncertainty reporting, and testing across imaging devices and populations.

### 4.4. Ensemble and Hybrid Models

Ensemble models combine multiple classifiers to improve robustness and reduce variance, helping most when base models make partially uncorrelated errors [11,82]. Bagging trains classifiers on bootstrap samples and combines them by voting or averaging, with random forests a common example. Boosting trains learners sequentially so later models correct earlier errors, as in XGBoost and LightGBM, used by Zhao et al. [83] for DR risk prediction in type 2 diabetes. Stacking trains a meta-learner on base-models’ outputs. Arora et al. [11] built an EfficientNet ensemble for DR on a Kaggle dataset. Ensembles usually outperform single models, but comparisons rarely use equal compute and are seldom tested on external data. This was confirmed by Matta et al. [84], who evaluated multi-disease fundus models across four populations. The study found that screening performance dropped on external data, and that a model trained on a single dataset will generalize worse, in comparison with one trained on several. This implies that a result measured on one benchmark cannot be assumed to hold once the camera or patient population changes.

A hybrid architecture combines complementary assumptions within one system. Selvin and Akila [85] chained GAN-based augmentation, ResNet101 features, and an XGBoost-LSTM classifier for DR. Though such multi-stage pipelines are harder to validate end-to-end. CNN-transformer designs are the most common fused form, using convolution for local features and self-attention for global context. Zhang et al. [86] illustrated this with TranSegNet for retinal OCT segmentation, which outperformed FCN, SegNet, U-Net, and TransUNet while keeping parameters low, though the result comes from the authors’ own curated OCT datasets rather than a test across imaging devices. Multimodal fusion extends the principle across imaging types. Gu et al. [87] showed a fundus–OCT fusion model outperforming single-modality baselines across seven conditions. However, the results were validated only across internal centers, since no public bimodal dataset yet exists. Such fusion mirrors how clinicians weigh multiple sources but sharpens the evaluation problem, since each added modality raises data and alignment demands. Also, few studies test whether the model holds when a modality is missing, which is a common case in primary care.

These gains carry a computational cost, since deep ensembles and hybrids increase memory use, latency, and energy consumption, limiting use in primary-care and community settings without high-end GPUs. Compression is therefore clinically relevant. Pruning removes redundant parameters, quantization reduces numerical precision, and knowledge distillation transfers a large teacher’s behavior to a smaller student.

The performance of these approaches ultimately depends on adequate dataset availability. The next section examines the datasets that support and constrain the methods reviewed in this section.

## 5. Classification and Segmentation Datasets

**Figure 2:**
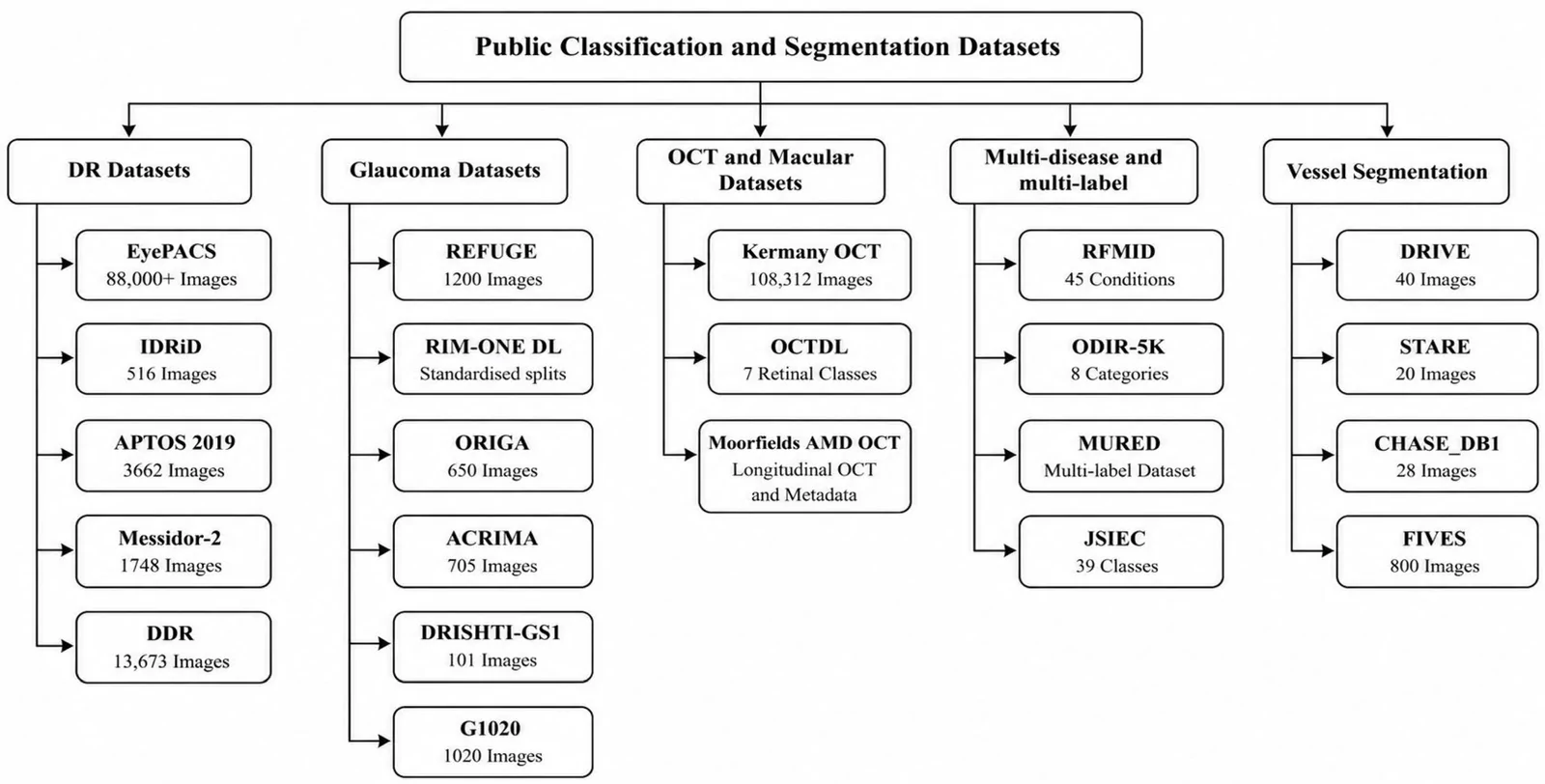
Public classification and segmentation datasets grouped by disease category [33,91,92,93,94,95,96,97]. Abbreviations: DR—Diabetic Retinopathy; IDRiD—Indian Diabetic Retinopathy Image Dataset; APTOS—Asia Pacific Tele-Ophthalmology Society; DDR—Dataset for Diabetic Retinopathy; REFUGE—Retinal Fundus Glaucoma Challenge; RIM-ONE DL—Retinal Images for Optic Nerve Evaluation, Deep Learning; ORIGA—Online Retinal Fundus Image Database for Glaucoma Analysis and Research; OCT—Optical Coherence Tomography; AMD—Age-related Macular Degeneration; RFMiD—Retinal Fundus Multi-Disease Image Dataset; ODIR-5K—Ocular Disease Intelligent Recognition; MuReD—Multi-Label Retinal Diseases Dataset; JSIEC—Joint Shantou International Eye Centre; DRIVE—Digital Retinal Images for Vessel Extraction; STARE—Structured Analysis of the Retina; CHASE_DB1—Child Heart and Health Study in England, Database 1; FIVES—Fundus Image Vessel Segmentation.

The datasets used to train and test a model determine how far its reported performance can be trusted. The major public datasets for eye disease detection are shown in Figure 2. Publicly available benchmark datasets have advanced research on DR, glaucoma, OCT-based macular disease, and vessel segmentation, whereas sparse or unavailable data continue to limit work on cataract, corneal disease, uveitis, and pediatric disorders [88]. Several dimensions determine whether reported performance can support claims of clinical utility. They are population diversity, acquisition and imaging device heterogeneity, disease severity distribution, annotation granularity, label reliability, external-validation suitability, licensing, and relevance to low-resource deployment.

A further consideration is the level at which labels are assigned, which may be image-level, eye-level, or patient-level [89]. The distinction matters because these units are nested and statistically dependent, since one eye contributes several images, and one patient contributes two correlated eyes. When this structure is disregarded and data are partitioned at the image level, images from the same patient may enter both the training and test sets. This will result in leaking the identity, thereby inflating reported performance. This was demonstrated directly for OCT classification by [90], where image-level splitting produced higher test accuracy than patient-level splitting on identical data. Patient-level partitioning is therefore a precondition for trustworthy evaluation rather than an optional refinement, yet many benchmark studies report neither the split level nor metrics aggregated to the clinically relevant unit. Label reliability compounds the concern, since reference standards are expert-graded and carry measurable disagreement, and datasets vary in whether they report inter-grader agreement or adjudicate conflicting labels. RFMiD, for instance, derives its labels through adjudicated consensus [91], whereas many datasets provide neither agreement statistics nor an adjudication protocol, leaving the achievable high performance undefined.

These limitations are magnified by a reliance on a small set of curated benchmarks that are rarely tested beyond their source distribution. Large population cohorts offer a route to external validation that remains unexploited, as discussed in Section 5.6. The subsections consider these where available, and Table 2 consolidates the datasets by disease coverage, sample size, modality, and annotation granularity.

### 5.1. Diabetic Retinopathy Datasets: EyePACS, IDRiD, Messidor, APTOS and DDR

DR has well-established public datasets for research in eye disease. EyePACS is one of the most widely used benchmarks, with more than 88,000 fundus images across five severity grades [98]. Alternatively, IDRiD provides image-level ICDR grades and pixel-level lesion annotations, making it central to lesion-localization research [92]. Also, Messidor-2 offers standardized diabetic-screening images [99], while APTOS 2019 adds more heterogeneous clinical contexts [100]. The popular DDR extends lesion-level benchmarking across 147 hospitals in 23 Chinese provinces, including a pixel-masked subset [93]. Together, these datasets support both image-level grading and lesion-level segmentation. However, differences in label quality, imaging protocol, and demographic coverage complicate direct comparison across these benchmark datasets.

### 5.2. Glaucoma Datasets: REFUGE, RIM-ONE DL, ORIGA, ACRIMA, DRISHTI-GS1 and G1020

Glaucoma datasets mainly support optic disc and cup segmentation, optic nerve head analysis, and image-level classification. The popular REFUGE provides optic-disc and optic-cup masks with clinical glaucoma labels for joint segmentation and classification [94]. The RIM-ONE DL combines earlier RIM-ONE versions into a deep-learning-oriented dataset with expert segmentations, standardized splits, and clinical labels [33]. Similar datasets such as ORIGA, ACRIMA, DRISHTI-GS1, and G1020 complement these with distinct sample and annotation characteristics, from image-level labels to expert optic-disc and optic-cup masks [101,102,103,104]. The popular benchmark dataset’s differences in sample size, class balance, resolution, and acquisition limit direct comparison, and their modest scale restricts assessment of generalization across populations and imaging devices.

### 5.3. OCT and Macular Disease Datasets: Kermany OCT, OCTDL, and Moorfields AMD OCT

Typically, OCT datasets support most eye disease detection tasks on AMD, DME, and drusen. The popular Kermany dataset provides 108,312 OCT B-scans across CNV, DME, drusen, and normal categories [18]. As a viable alternative, the OCTDL extends this to seven retinal classes acquired on a single imaging device, which improves internal uniformity but limits cross-device evaluation [105]. Also, the Moorfields AMD dataset links longitudinal records from patients receiving anti-VEGF treatment, thus supporting treatment–response and longitudinal modelling rather than basic-level image diagnosis [106,107]. The popular dataset’s differing class definitions, imaging devices, and institutional settings confound direct comparison. Also, models trained on a single imaging device or center may capture acquisition-specific characteristics that do not transfer well across different imaging devices or clinical settings.

### 5.4. Multi-Disease and Multi-Label Datasets: ODIR-5K, RFMiD, MuRED, and JSIEC

Multi-disease datasets more closely reflect eye disease screening, since a single patient or image may exhibit several concurrent diseases, whereas they differ considerably in unit of analysis, labeling structure, image sources, and/or coverage of uncommon conditions. The popular ODIR-5K provides bilateral fundus images from 5000 patients across eight categories, acquired on several commercial cameras [108]. However, the dataset imbalance between common and uncommon categories makes reliable detection of minority labels difficult. A viable alternative is the popular RFMiD, which comprises 3200 annotated images with 46 classes, adjudicated by consensus. Thus, it provides broad pathology coverage [91]. Also, MuReD integrates ARIA, STARE, and RFMiD into 2208 images with 20 labels. The dataset admirably reduces some imbalance but requires careful duplicate control [109]. Furthermore, the public JSIEC subset offers 1000 images across 39 categories. The dataset is highly valuable for wide taxonomies, but its size per category for independent large-model training is a notable concern [110]. While these publicly available benchmark datasets represent a shift toward broader, more representative screening, their limitations lie in class imbalance concerns, inconsistent label definitions, overlapping source images, and small-size minority class samples. Thus, strong aggregate performance may conceal poor sensitivity for clinically important minority classes.

### 5.5. Retinal Vessel Segmentation Datasets: DRIVE, STARE, CHASE_DB1, and FIVES

Vessel segmentation datasets provide pixel-level reference annotations for evaluating vascular extraction. They derive measures such as caliber, tortuosity, and vascular density, relevant to DR, hypertensive change, glaucoma, and systemic vascular disorders. DRIVE, STARE, and CHASE_DB1 remain widely used due to their expert annotations, which allow comparison with earlier methods. However, their small sizes make them vulnerable to overfitting and provide limited evidence across diseases, imaging devices, and populations [96,111,112]. FIVES offers a stronger basis, with 800 high-resolution images spanning normal, DR, AMD, and glaucoma, each with a disease label, quality assessment, and pixel-level vessel mask [97]. Recently, DRIVE, CHASE_DB1, and FIVES have also been used as annotated source datasets for domain-adaptive vessel segmentation, demonstrating their relevance beyond conventional within-dataset benchmarking [113]. Nevertheless, dataset-specific differences in image quality and acquisition characteristics can still influence segmentation performance.

### 5.6. Population-Scale and Longitudinal Cohorts

Beyond the public benchmarks above, large population cohorts form a distinct class of resources. The most prominent is the UK Biobank, with fundus photography and OCT acquired from roughly 130,000 participants and linked to systemic health records and long-term outcomes [114]. It is mainly useful in pretraining foundation models on large retinal datasets, deriving imaging biomarkers against systemic disease, and supporting external validation across a broad population rather than in benchmark classification. The Moorfields anti-VEGF records add a longitudinal clinical cohort for treatment–response and progression modelling [106,107]. These cohorts differ from the open benchmarks in two respects. They provide repeated imaging and linked outcomes rather than single cross-sectional grades, and access is controlled through application rather than open download, which constrains reproducibility but improves clinical and demographic breadth. Their main limitation for detection research is that disease labels are derived from health-record coding rather than standardized image grading, so label granularity is coarser than in purpose-built benchmarks.

Table 2 shows progression from small, disease-specific datasets toward larger multi-disease and multi-label resources. As shown in Table 2, extensive work has recently been done towards multi-label and multi-disease data capture and annotation. However, three limitations persist, namely: (1) severe and uncommon conditions are frequently represented by few images, weakening class-specific evaluation; (2) demographic characteristics such as age, sex, ethnicity, and geographical origin are often incompletely reported; and (3) many multi-disease datasets remain strongly imbalanced. These limitations restrict direct comparison and weaken evidence of generalizability across populations, institutions, and imaging devices. They also reach into evaluation itself, since the class imbalance running through these datasets distorts the metrics most often reported. Positive and negative predictive values depend on disease prevalence, so strong sensitivity and specificity on a balanced benchmark can still translate into poor predictive value at the low prevalence typical of community screening. Reporting should therefore extend beyond accuracy and AUC to the area under the precision–recall curve (AUPRC), sensitivity at a fixed clinically acceptable specificity, decision curve analysis, calibration, and the Brier score.

The structure and labeling of these datasets, whether image-level grades, multi-label annotations, or pixel-level masks, determine how detection can be formulated as a learning problem. The next section examines the classification tasks supported by these datasets.

**Table 2:** Summary of publicly available datasets for AI-based eye disease detection and segmentation reviewed in Section 5.

Dataset	Disease or Target	Size	Modality	Annotation Granularity	Reference
EyePACS/Kaggle DR	DR, five severity grades	88,702 images	Fundus	Image-level severity grade	[115]
IDRiD	DR grading and lesion segmentation	516 images	Fundus	Image-level grade and pixel-level lesion masks	[92]
Messidor-2	DR grading	1748 images	Fundus	Image-level severity grade	[99]
APTOS 2019	DR grading	3662 images	Fundus	Image-level severity grade	[100]
DDR	DR grading and lesion segmentation	13,673 images; 757 annotated	Fundus	Image-level grade, boxes, and pixel-level masks	[93]
REFUGE	Glaucoma assessment	1200 images	Fundus	Optic-disc/cup masks and glaucoma label	[94]
RIM-ONE DL	Glaucoma assessment	485 images	Fundus	Glaucoma label and manual OD/OC masks	[33]
G1020	Glaucoma assessment	1020 images	Fundus	Glaucoma label and OD/OC annotations	[101]
ORIGA-light	Glaucoma and ONH analysis	650 images	Fundus	Clinical labels and OD/cup annotations	[104]
ACRIMA	Glaucoma classification	705 images	Fundus	Image-level glaucoma label	[102]
DRISHTI-GS1	Optic-disc and cup segmentation	101 images	Fundus	Expert pixel-level OD/OC masks	[103]
Kermany OCT	CNV, DME, drusen, normal	108,312 images	OCT	Image-level four-class label	[18]
OCTDL	AMD, DME, ERM, RAO, RVO, VID, normal	2064 images	OCT	Image-level seven-class label	[105]
ODIR-5K	Eight broad ocular categories	5000 patients; paired eye	Fundus	Patient-level bilateral multi-label	[116]
RFMiD	Retinal diseases and abnormalities	3200 images	Fundus	Image-level multi-label for 45 plus normal	[91]
MuReD	Multiple retinal diseases	2208 images; 20 labels	Fundus	Image-level multi-label	[109]
JSIEC public subset	39 fundus diseases and conditions	1000 images	Fundus	Image-level 39-class label	[110]
DRIVE	Retinal vessel segmentation	40 images	Fundus	Pixel-level vessel masks	[96]
STARE	Retinal vessel segmentation	20 images	Fundus	Two manual vessel masks per image	[112]
CHASE_DB1	Pediatric retinal vessel segmentation	28 images	Fundus	Two manual vessel masks per image	[111]
FIVES	Vessel segmentation across normal, DR, AMD, glaucoma	800 images	Fundus	Vessel masks, disease labels, quality scores	[97]
UK Biobank (Eye and Vision)	Population cohort; DR, glaucoma, AMD, and systemic disease	~130,000 participants	Fundus and OCT	Linked health-record diagnoses; controlled access	[114]

Abbreviations: CNV—Choroidal Neovascularization; DME—Diabetic Macular Edema; AMD—Age-related Macular Degeneration; ERM—Epiretinal Membrane; RAO—Retinal Artery Occlusion; RVO—Retinal Vein Occlusion; VID—Vitreomacular Interface Disease; OD—Optic Disc; OC—Optic Cup.

## 6. Classification Tasks in Eye Disease Detection

**Figure 3:**
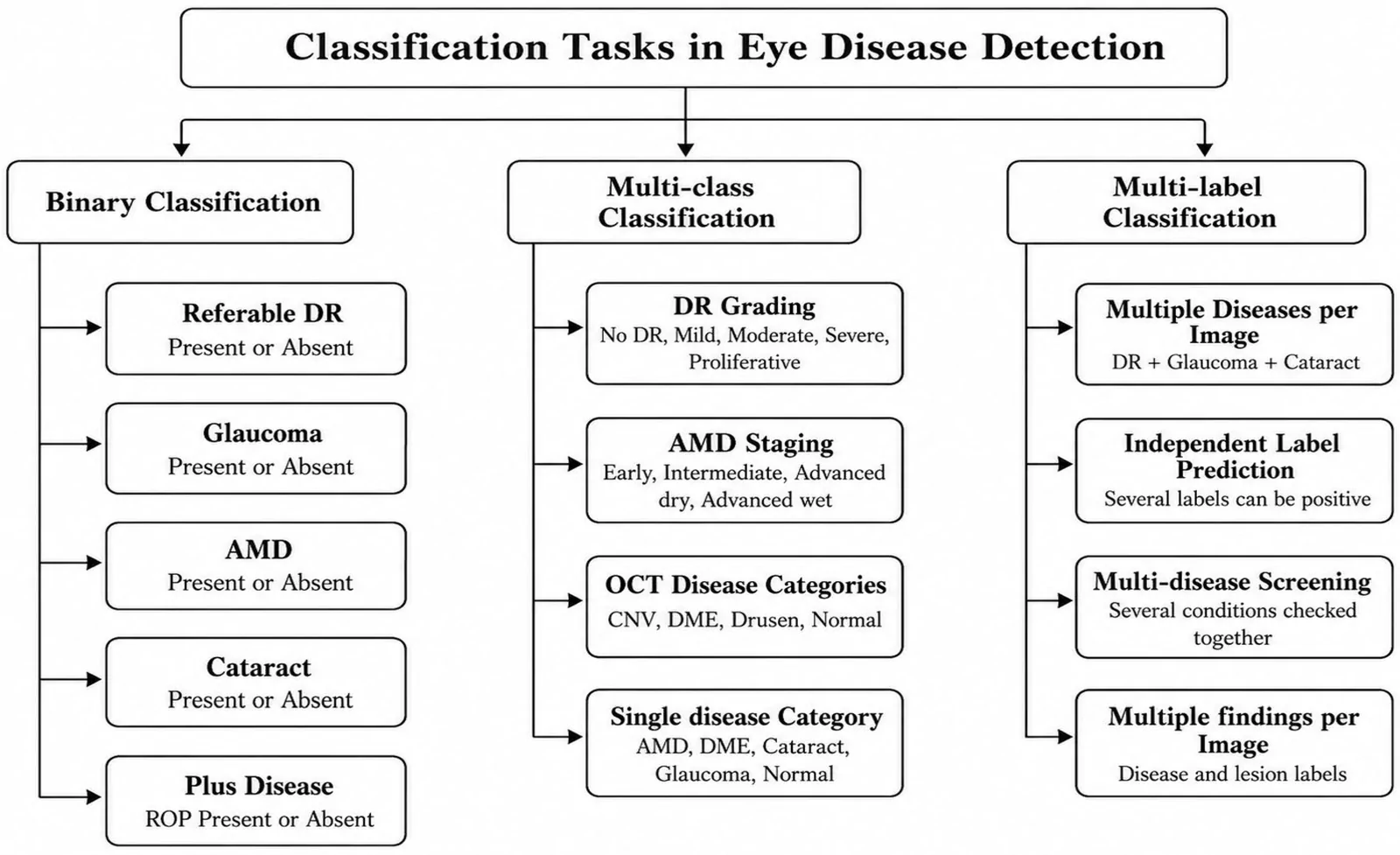
Classification tasks in eye disease detection, showing binary, multi-class, and multi-label approaches [17,117,118]. Abbreviations: DR—Diabetic Retinopathy; AMD—Age-related Macular Degeneration; OCT—Optical Coherence Tomography; CNV—Choroidal Neovascularization; DME—Diabetic Macular Edema; ROP—Retinopathy of Prematurity.

Eye disease detection can be framed as binary, multi-class, or multi-label classification. Binary classification distinguishes disease from healthy or referable from non-referable. Multi-class classification assigns each image to one of several mutually exclusive categories, such as a severity grade. Multi-label classification detects multiple coexisting diseases in the same image, reflecting the frequent co-occurrence of retinal conditions [17,117,118]. Figure 3 illustrates the different classification tasks for eye disease detection.

### 6.1. Binary Classification

Binary classification is the most common task, separating diseased from healthy or referable from non-referable diseases. It typically uses sigmoid outputs and binary cross-entropy loss, evaluated using ROC curves, AUC, sensitivity, and specificity. Gulshan et al. [17] developed an influential automated system for referable DR. Akhtar et al. [119] extended this with RSG-Net, which jointly performed binary DR detection and four-stage grading, showing that multitask learning can improve screening sensitivity. Binary classification also extends to neonatal screening. Brown et al. [51] trained a CNN to diagnose plus disease, the vascular dilation and tortuosity signaling treatment-requiring ROP. They achieved an accuracy comparable to or better than expert graders. This is clinically valuable, since plus-disease diagnosis is highly subjective. That same subjectivity, however, weakens the reference standard, since a model trained on expert consensus inherits the variability it is meant to reduce. False negatives carry substantial clinical risk, hence screening systems should prioritize sensitivity even at some cost to specificity. Binary classification is thus a useful entry point, but its two-category structure cannot capture severity, progression, or treatment-relevant heterogeneity.

### 6.2. Multi-Class Classification

Multi-class classification distinguishes three or more mutually exclusive categories. It supports severity grading such as five-level DR and single-label differential diagnosis. Kermany et al. [18] classified OCT scans into CNV, DME, drusen, and normal, while other studies assign a single label per fundus image across conditions such as AMD, cataract, and glaucoma [105]. These models generally use softmax outputs with categorical cross-entropy loss. They are evaluated using a confusion matrix, per-class sensitivity and specificity, and macro- or weighted F1. The main limitation is class imbalance, since screening datasets contain many normal or mild cases and few severe ones. Chlopowiec et al. [120] demonstrated on ODIR, where minority classes showed reduced sensitivity without imbalance correction. Common mitigations include SMOTE, undersampling, class-weighted and focal losses, and targeted augmentation [121]. Fusion architectures can improve multi-class fundus classification over single-model baselines [122]. Nevertheless, mutual exclusivity limits clinical realism, since many patients have co-occurring diseases that a single-label model cannot represent.

### 6.3. Multi-Label Classification

Multi-label classification addresses co-occurring disease by allowing several conditions to be recognized in the same image. Predicting each disease is done independently through sigmoid outputs with per-label binary cross-entropy loss [123]. Evaluation must therefore capture both label-level and whole-prediction performance, using per-label AUC, sensitivity, and specificity alongside micro- and macro-F1, Hamming loss, and exact match ratio. Since a model can score well on common labels while failing the rare ones, a single accuracy figure is not appropriate. ODIR-5K and RFMiD are the principal benchmarks, with RFMiD providing 3200 images across 46 classes, that is, 45 disease labels plus normal.

Recent work has concentrated on imbalance and inter-disease dependency, but progress is measured almost entirely on benchmark data and on the labels easiest to learn. Elsayed and Rushdi [118] embedded a graph-theoretic prior over inter-disease relationships within RFMiD. Their model covered 28 of the RFMiD categories rather than the full 45, excluding precisely the rare diseases a dependency prior should help most. Iqbal et al. [124] combined SMOTE, focal loss, and Grad-CAM on ODIR, RFMiD, and IDRiD to show that imbalance mitigation and explanation can be pursued together. But on curated public benchmarks, this speaks to feasibility rather than performance under clinical variation. Collectively, the methodological tools for multi-label eye disease classification now exist but have not been shown to deliver on the long tail of rare diseases or across the devices and populations of real screening. This leaves generalizability across imaging devices, patient populations, and healthcare settings largely untested.

In summary, binary, multi-class, and multi-label classification form a range from simplicity to clinical realism. Binary models provide efficient screening, multi-class models add diagnostic granularity, and multi-label models most closely reflect concurrent disease, at the cost of greater methodological complexity, data demands, and susceptibility to imbalance. The choice of task should therefore be guided not by benchmark performance alone but by whether a model holds up on rare conditions and across populations in its deployment context. Table 3 consolidates representative studies across the methodological families reviewed in Section 4 and the classification tasks reviewed in this section.

## 7. Segmentation Approaches in Eye Disease Detection

Segmentation complements classification by producing pixel-level anatomical or pathological maps [129]. It is clinically important, since cup-to-disc ratio, retinal vascular caliber, lesion burden, and drusen extent all require accurate structural delineation before supporting grading or risk stratification. Segmentation functions as an interpretable bridge between image appearance and clinically measurable biomarkers. The main categories are retinal vessel segmentation, optic disc and cup segmentation, and lesion segmentation. Each addresses a distinct anatomical target [130].

### 7.1. Retinal Vessel Segmentation

Retinal vessel segmentation supports DR, hypertensive retinopathy, glaucoma, and systemic vascular-risk assessment. The baseline is U-Net, with its encoder–decoder structure and skip connections preserving spatial detail [74]. The authors in [131] later proposed Attention U-Net to suppress irrelevant background and improve localization of thin vessels [131]. Full-scale guided networks combine multi-scale representation with attention-guided filtering to preserve fine capillaries [132]. These models are typically benchmarked on DRIVE, STARE, CHASE_DB1, and FIVES, whose modest sizes reflect the cost of expert pixel-level annotation. That reliance is itself a limitation, since gains on a handful of datasets sharing acquisition characteristics do not establish that a model segmenting capillaries well on DRIVE will transfer to the scanners and image quality of an unscreened population.

### 7.2. Optic Disc and Cup Segmentation

Optic disc and cup segmentation supports glaucoma screening through cup-to-disc ratio estimation. Fu et al. [133] proposed M-Net, which jointly segments disc and cup. In the study, polar transformation was used to represent their circular boundaries, reducing anatomically implausible outputs such as a cup extending beyond the disc. Recent literature studies typically deploy REFUGE and RIM-ONE DL as major benchmarks [33,94]. It is also noted that studies increasingly evaluate segmentation and glaucoma classification jointly rather than independently. The task is well studied but not solved, since the optic cup has an indistinct boundary that even expert annotators delineate inconsistently. This label variability places a ceiling on achievable accuracy and propagates directly into the cup-to-disc ratio. Therefore, a segmentation model can match its reference masks while inheriting their uncertainty. The persistence of this problem, despite architectural progress, indicates that the limiting factor in disc-and-cup segmentation is annotation reliability rather than network design.

### 7.3. Lesion Segmentation

Lesion segmentation targets small pathological structures that determine severity, such as microaneurysms, hemorrhages, and exudates in DR. Wan et al. [134] proposed EAD-Net, an encoder–attention–decoder architecture that improved sensitivity and F1-score over U-Net on e-ophtha and IDRiD. This task intensifies class imbalance, since lesion pixels occupy only a small fraction of each image, and specialized losses, multi-scale features, and attention are essential. That same imbalance inflates pixel-level accuracy and specificity, so headline figures can appear strong while the clinically decisive quantity, sensitivity to the smallest and rarest lesions, remains harder to achieve and less consistently reported.

Across all three tasks, segmentation architectures are moving from purely convolutional encoder–decoders towards transformer-augmented hybrids. TransUNet combines convolutional localization with transformer-based global context and has been adapted to retinal segmentation [135]. Weakly and self-supervised methods address the annotation bottleneck by learning from image-level labels, attribution maps, or unlabeled images. A further direction unifies segmentation and classification. Li and Tao [136] proposed V-MNet, a vessel-guided network that jointly performs vessel segmentation and multi-disease classification. The segmentation branch was used as spatial attention for classification, reporting a vessel Dice of 0.831 and AUC of 0.985 and a multi-disease mean AUC of 0.978 and F1 of 0.935, with ablation gains over single-task counterparts. The design encodes an explicit, inspectable link between vascular structure and disease rather than leaving it implicit in shared weights. This aligns the model with how clinicians’ reason for vascular disease, though the gains remain single-study and single-benchmark and require external validation. Collectively, classification and segmentation now form an integrated ecosystem. Classification provides an image-level decision, while segmentation identifies the spatial biomarkers that support interpretation of that decision. Both share advances in CNNs, transformers, attention, and self-supervision, while segmentation faces the major bottleneck of pixel-level annotation scarcity. Table 4 summarizes representative segmentation studies.

Together, these approaches produce predictions and structural maps that support automated detection of eye diseases. Whether these outputs can be trusted in clinical use depends on the interpretability, which is discussed in the next section.

## 8. Explainable Artificial Intelligence

High predictive performance does not establish clinical trust, so explainable AI (XAI) addresses this limitation by making these decisions auditable and clinically interpretable [137]. Four categories of XAI are relevant, namely post hoc attribution, attention-based explanation, concept-based explanations, and counterfactual explanation. These categories are often treated as a single interpretive toolkit, yet each addresses a distinct question about the model, and that difference bears directly on what an explanation can legitimately certify [138]. Post hoc attribution methods include gradient-based maps such as Grad-CAM [139] and perturbation-based methods such as LIME [140] and SHAP [141], which identify the image regions that most influenced a prediction after it has been made. Attention-based explanation, exposed by vision transformers and attention modules, reports where the model allocated weight during inference [142,143]. Concept-based explanation tests whether human-defined clinical concepts, rather than incidental image statistics, govern the prediction [144]. Counterfactual explanation identifies the minimal change to an image that would reverse the decision, thereby exposing the model’s effective decision boundary [145]. Attribution and attention reveal only where a model attends, whereas concept-based and counterfactual methods probe what determines the decision, so the former can appear persuasive while the causal basis of a prediction remains unverified.

Grad-CAM, a gradient-based method, differentiates a class score with respect to convolutional feature maps and produces a heatmap of influential regions [139]. Applied to DR, glaucoma, AMD, and cataract, Grad-CAM and Grad-CAM++ highlight recognizable features such as microaneurysms, hemorrhages, optic-disc cupping, and drusen [146]. These heatmaps are visually persuasive but not necessarily correct. Grad-CAM output shifts with the chosen target layer, varies across identical training runs, and can settle on anatomically plausible regions that play no part in the decision. A heatmap a clinician judges as correct is therefore not evidence that the model reasoned correctly. Shyamalee et al. [128] integrated Grad-CAM into a glaucoma system and reported alignment with expert optic-disc annotations. Pathmakumara and Perera [147] localized DenseNet121 glaucoma explanations to the optic nerve head. Since the optic nerve head is the primary site clinicians examine, an explanation that localizes these matches expectations regardless of whether the model relied on it. An identical heatmap can arise from genuine detection of optic-disc cupping or from reliance on an incidental correlate such as a scanner signature or labeling pattern. Since agreement with expert annotation cannot distinguish these, it is the weakest available evidence of interpretability.

Perturbation-based methods such as LIME and SHAP explain any model by measuring how input changes move the prediction [140,141]. This makes them applicable to traditional ML, ensembles, tabular models, and CNNs. Aldughayfiq et al. [126] applied LIME and SHAP to an InceptionV3 retinoblastoma classifier. They found explanations consistent with clinical findings, though evidence from a single binary task on 800 images limits generalizability. Hybrid frameworks combining Grad-CAM localization with SHAP-style importance improve the presentation of an explanation without establishing its correctness, since their value depends on the faithfulness of each component, and neither has been independently validated in this domain [147]. Attention-based explanation is more integrated with architecture. ViTs expose attention across patches, and studies report correspondence with diseased regions in fundus and OCT images [142,143], while CBAM and SE modules provide auxiliary attention maps [81]. Reported correspondence does not establish that attention drove the prediction, since a model can attend to a region without relying on it. Therefore, a map concentrated on a plausible lesion is a hypothesis about model behavior rather than confirmation of it. The convergence of post hoc attribution and built-in attention indicates that explainability should be treated as a design requirement, with explanations presented in formats clinicians can interpret without machine-learning expertise [137]. Across all three families, the eye disease XAI evidence base is narrow, most reported successes rest on a single study or disease, and no method has been shown to recover a model’s true decision basis rather than a plausible proxy. Current methods therefore demonstrate capability rather than validated faithfulness.

## 9. Research Gaps and Future Directions

Despite rapid progress in AI-based eye disease detection, structural gaps continue to limit clinical translation and public-health impact [148]. Notably, these barriers are not isolated technical shortcomings but recur across clinical readiness, data representation and equity, validation under real-world imbalance, and interpretability. This section synthesizes these gaps and identifies where progress is most needed to move AI-based eye disease detection from retrospective benchmark success toward clinically reliable deployment.

### 9.1. Clinical Readiness, Calibration, and Decision Thresholds

Clinical readiness cannot be assessed using discrimination metrics such as accuracy or AUC alone. AI-based eye disease analysis systems should also report calibration, uncertainty, decision thresholds, failure cases, subgroup performance, and referral consequences. Calibration curves, expected calibration error, Brier score, as well as threshold-specific sensitivity and specificity matter particularly in screening, since poorly calibrated probabilities can distort referral decisions even when AUC remains high. These thresholds are not universal, since the cost of a missed case and the action it triggers differ by disease, so they must be chosen per disease and re-tuned to the prevalence of each deployment setting. Deployment workflows should also incorporate uncertainty estimation. Monte Carlo dropout, deep ensembles, test-time augmentation, and conformal prediction can flag low-confidence cases for human review. In low-resource settings, these triage mechanisms may matter as much as raw accuracy, since they reduce the chance that ambiguous or poor-quality images are processed without clinical oversight. Table 5 shows a clinical-readiness checklist for AI-based eye disease detection systems.

### 9.2. Data Representation and Equity

The most visible imbalance in recent literature is in terms of which diseases and populations the deployed dataset(s) represent. Evidently, DR has attracted disproportionate attention because mature public datasets made it an early and tractable target. However, cataract, which is the leading cause of blindness globally, along with corneal disease, uveitis, and most pediatric conditions, remains underrepresented and less analyzed. While it is worth mentioning that ODIR-5K and RFMiD include cataract and corneal labels, their class distributions remain highly imbalanced [123]. Data availability has therefore shaped the research agenda, since well-resourced disease areas attract more models, citations, and benchmark refinement, while other clinically important conditions remain methodologically underdeveloped.

The same concentration appears geographically. Research clusters in high-income settings, while the populations, imaging conditions, and health-system constraints of many low- and middle-income countries remain underrepresented despite their disproportionate burden of preventable blindness [1]. Models developed without adequate representation of Sub-Saharan African, South Asian, or Latin American data may not generalize reliably to those settings. Federated learning offers a practical route to multi-country training without transferring raw patient data [12,77], but the technical mechanism is only half the solution. Future work should pair large, clinically annotated datasets for underrepresented diseases and regions with equitable partnerships that give those regions a central role in dataset design, validation, and deployment governance.

### 9.3. Validation and Severe-Disease Performance

A further barrier is whether benchmark performance transfers to practice, since many deep learning systems lose accuracy across different imaging devices, acquisition protocols, populations, and workflows [149]. External validation is usually used in this regard, yet it is routinely reported as a single construct, with one external test set offered as evidence of general robustness [149,150]. The four forms of external validation such as temporal, geographic, device-based, and population-based validation are not equivalent, and each exposes a failure the others can miss. Temporal validation guards against drift as equipment and case mix change over time, so a model split only contemporaneously can appear stable while decaying silently in use. Geographic validation tests it at new sites and health systems, revealing dependence on site-specific acquisition and referral patterns [150]. Device-based validation tests it on cameras or scanners absent during training, where differences in resolution, color, and noise alone can shift predictions, and it is the form most often omitted. Population-based validation tests it on different demographic and disease-prevalence distributions, where predictive value and subgroup fairness can both deteriorate, which makes it the most consequential for equity [149]. Passing one axis therefore licenses no claim about the others, yet reviews and regulatory submissions often treat a single external cohort as sufficient. Prospective clinical validation and randomized trials remain the gold standard, since only a live pipeline exposes the interaction of model, operator, image quality, and workflow that retrospective testing holds fixed.

The gap between retrospective and prospective evidence is the clearest demonstration of this. The pivotal trial of IDx-DR validated the system prospectively in primary-care clinics and met prespecified sensitivity and specificity, which supported its regulatory clearance [26]. A diabetic retinopathy model with strong retrospective accuracy, by contrast, degraded once deployed prospectively in clinics whose image quality and workflow differed from its development data, where images were rejected at the point of care and patients were sent away [80]. The difference between the two was not the algorithm but whether evaluation reproduced deployment conditions.

The standard offline training, held-out-testing paradigm cannot detect this, since benchmark samples may not represent real-clinic prevalence, image quality, or demographics. Class imbalance sharpens the problem, since screening datasets contain many normal or mild cases but few severe, referral-critical examples. Therefore, a model can post high aggregate accuracy while underperforming on precisely the cases that matter most for clinical safety [151].

Mitigation strategies address different failure modes and are not interchangeable. Loss-based methods such as focal loss and label-distribution-aware margin operate on real images and preserve anatomical fidelity, reweighing the optimization objective rather than synthesizing data [151]. Their limit is that they cannot expand minority-class visual diversity. Generative approaches can expand that diversity, but their clinical value depends on preserving fine lesion morphology. Zhou et al. [152] showed that DR-GAN generates high-resolution fundus images with controllable lesion placement when lesion masks explicitly constrain synthesis. Nderitu et al. [153] found that an unconstrained diffusion generator produced images that graders consistently assessed as showing no retinopathy when conditioned to generate mild DR, since it failed to reproduce microaneurysms reliably. Generative augmentation is therefore most defensible when lesion-level structure is enforced, and loss-based methods remain safer when lesion fidelity cannot be verified. The more durable solution is prospective enrichment of annotated severe-disease cohorts, particularly for proliferative DR, advanced glaucoma, and wet AMD. This will help models learn high-risk phenotypes directly rather than relying on statistical correction of imbalanced benchmarks. Prospective, multi-center validation on demographically diverse cohorts, with reporting standards such as CONSORT-AI treated as a minimum rather than an addendum, should become the default rather than the exception [154].

### 9.4. Explainability and Clinician Trust

Even well-validated models require clinician trust before they can influence care. Many eye disease systems provide limited transparent evidence for their predictions, which makes them difficult to audit [155]. XAI methods such as Grad-CAM highlight image regions that contribute to a decision and have been applied to DR, glaucoma, and AMD [139,146]. However, they are typically applied as post hoc visual explanations rather than integrated into clinically legible workflows [156]. Abbas et al. [157] found that integrating explanation methods such as LIME can improve transparency and clinician trust in automated eye disease prediction, yet trust should rest on validated explanatory quality, not visual plausibility alone.

Current XAI evaluation is weak, since it often relies on qualitative inspection by a few clinicians. This is subjective, hard to reproduce, and insufficient for multi-site deployment. Eye disease XAI should instead report quantitative explanation metrics such as localization accuracy through intersection-over-union or Dice overlap with expert lesion masks, faithfulness through the drop in model confidence when highlighted regions are occluded, and stability under small input perturbations [158]. These metrics are feasible, since datasets such as IDRiD already provide pixel-level annotations. Interpretability should become a methodological standard, evaluated with the same rigor as classification performance, and explanations should be presented in formats clinicians can review without specialist machine-learning expertise.

Finally, these four gaps share a common root. Current evidence is generated under conditions systematically easier than deployment, whether in the populations sampled, the imbalance tolerated, the devices tested, or the explanations accepted. The unifying priority is therefore to reduce the gap between evaluation conditions and deployment conditions, so that benchmark success becomes a reliable predictor of clinical performance rather than a weak proxy for it. These gaps are not closing evenly across tasks. Referable diabetic retinopathy screening is closest to clinical readiness, supported by prospective and regulatory evidence, whereas multi-label recognition, rare-disease detection, and cross-device deployment remain largely at the research stage.

### 9.5. Limitations of the Review Study

This review has a few limitations. First, it is a structured narrative review rather than a registered systematic review, so it does not claim exhaustive retrieval, predefined inclusion criteria, or formal risk-of-bias assessment, and selection reflects the authors’ judgement of representativeness. Second, several areas surveyed, including foundation models, federated learning, and ViTs, are evolving rapidly, so the findings represent a snapshot that later work may extend or supersede. Third, the reported performance values are drawn from heterogeneous studies and are not directly comparable, since datasets, preprocessing, validation splits, disease prevalence, and metrics vary widely. Comparative statements should therefore be read as indicative of broad trends rather than head-to-head benchmarks.

## 10. Conclusions

AI-based eye disease detection has progressed from experimental image classification to a broad methodological field spanning traditional ML, deep neural networks, transformers, segmentation models, ensembles, federated learning, and foundation models. This reflects growing clinical demand for scalable systems that support early detection of vision-threatening disease, particularly where specialist services are limited. The main challenge is no longer whether eye images can support automated diagnosis, but whether these systems can maintain accuracy, interpretability, efficiency, fairness, and clinical reliability beyond controlled benchmark environments.

This review examined the major methodological approaches and clinical-use frameworks in AI-based eye disease detection, including feature-based classifiers, transfer-learned CNNs, U-Net-derived segmentation, attention mechanisms, ViTs, ensemble and hybrid architectures, explainable AI, federated learning, and foundation models. Binary, multi-class, and multi-label classification, and the datasets and evaluation practices that shape model development were also examined. Across these areas, consistent limitations exist. This includes high reported performance, which often depends on small datasets, limited external validation, incomplete demographic reporting, and inadequate representation of severe, rare, or under-studied eye diseases.

CNN-based transfer learning remains the established practical approach, while transformer-based and foundation-model methods are gaining importance for their ability to model broader retinal context and reduce dependence on large task-specific training sets. Interpretability methods have advanced in parallel yet remain visually persuasive rather than demonstrably faithful. Therefore, explainability in this field is still a demonstrated capability rather than a validated one. Future progress should therefore be judged not by small improvements on established benchmarks but by evidence of clinical reliability and transferability. Priority should be given to prospective multi-center validation, demographically and geographically representative datasets, improved representation of severe and rare disease, calibrated and interpretable predictions, transparent reporting, computationally efficient deployment, and federated or collaborative model development in low- and middle-income settings. Overall, AI-based eye disease detection has reached a high level of methodological development, but its clinical translation remains incomplete. Its long-term value will depend on improving diagnostic access, reliability, clinician trust, and equity across varied healthcare environments.

## Figures and Tables

**Table 1:** Search and selection approach for the narrative review.

Review Item	Details Included
Databases and repositories	IEEE Xplore, PubMed/MEDLINE, ScienceDirect, Scopus, arXiv
Main search concepts	Eye disease terms combined with machine learning, deep learning, ViT, XAI, segmentation, OCT, and fundus imaging terms
Primary period	2020–2026, with a few earlier foundational works included
Included sources	Journal articles, conference papers, dataset papers, and selected preprints with traceable bibliographic details
Excluded sources	Non-verifiable records, non-eye disease studies, and papers without adequate methodological or evaluation detail
Synthesis method	Narrative grouping by disease, modality, model family, task type, dataset, validation strength, and translational barrier

**Table 3:** Synthesis of representative studies across methodological families and classification tasks reviewed in Section 4 and Section 6.

Study	Method	Dataset	Task	Key Reported Result	Limitation
Gulshan et al. [17]	Deep CNN (Inception-v3)	EyePACS, Messidor-2	Binary referable-DR screening	Sensitivity/specificity matching US board-certified ophthalmologists	Retrospective, single-country cohort; no prospective validation
Kermany et al. [18]	Transfer-learned CNN	Kermany OCT	4-class OCT classification	96.6% accuracy	Single-institution OCT device; four categories only
Keerthiveena et al. [125]	BPSO wrapper + SVM	Ocular feature dataset	Feature selection + glaucoma/DR classification	Improved feature-selection efficiency and accuracy	Wrapper search adds computational overhead vs. filter methods
Aldughayfiq et al. [126]	InceptionV3 + LIME/SHAP	400 retinoblastoma/400 control	Explainable retinoblastoma diagnosis	Consistent SHAP/LIME explanations vs. clinical findings	Small, rare-disease dataset; limited statistical power
Li et al. [19]	Swin Transformer V2	OCT2017, OCT-C8	Multi-fundus OCT classification	99.9% (OCT2017); 99.5% (OCT-C8)	Near-ceiling accuracy on curated benchmarks; external validity untested
Zhang et al. [86]	CNN encoder + ViT hybrid	Kermany OCT	Retinal OCT segmentation	Outperforms U-Net and standalone ViT baselines	Single OCT dataset; cross-device generalization untested
Chetoui & Akhloufi [12]	Federated ViT	4 distributed institutions	DR detection	Competitive with centralized training on pooled data	Four sites only; scalability untested
Zhou et al. [58]	Self-supervised masked-autoencoder foundation model	1.6M unlabeled retinal images	DR/AMD/systemic prediction	Outperforms ImageNet-pretrained models; fewer labels needed	Pre-training corpus demographics not fully disclosed
Zhang et al. [127]	RETFound fine-tuning	Community screening, China	Fundus disease screening	>15% sensitivity/specificity gain vs. commercial tools	Validated within a single national healthcare system
Shyamalee et al. [128]	Segmentation + classification + Grad-CAM	Fundus images	Explainable glaucoma identification	Improved reported clinician confidence	Explainability assessed qualitatively; no powered clinician study
Hussein et al. [69]	ANN + DWT + MobileNetV2	Multi-disease fundus dataset	Multi-class (DR, cataract, glaucoma)	98.2% peak accuracy	Single-benchmark evaluation; external generalizability not assessed
Akhtar et al. [119]	Custom CNN from scratch	Messidor-1	Binary + 4-class DR grading	High accuracy on both heads	Single, small dataset; no cross-dataset testing
Iqbal et al. [124]	SMOTE + focal loss + Grad-CAM	ODIR, RFMiD, IDRiD	Multi-label classification + interpretability	State-of-the-art multi-label performance	Combines techniques without isolating each contribution
Gholami et al. [77]	Self-supervised domain-adaptive federated learning	OCT, multi-institution	Eye disease classification	AUC improved ≥10% vs. locally trained models	Federated simulation; real deployment not demonstrated

Abbreviations: BPSO—Binary Particle Swarm Optimization; LIME—Local Interpretable Model-agnostic Explanations; SHAP—SHapley Additive exPlanations; ViT—vision transformer; ANN—Artificial Neural Network; DWT—Discrete Wavelet Transform; MobileNetV2—Mobile Neural Network, Version 2; Grad-CAM—Gradient-weighted Class Activation Mapping; SMOTE—Synthetic Minority Oversampling Technique; AUC—Area under the Receiver Operating Characteristic Curve; EyePACS—Eye Picture Archive Communication System.

**Table 4:** Summary of representative segmentation studies, methods, datasets, and reported limitations.

Study	Method	Dataset	Task	Result	Limitation
Ronneberger et al. [74]	U-Net (encoder–decoder with skip connections)	General biomedical; adapted to fundus	Foundational vessel/structure segmentation	Outperformed prior sliding-window CNN	Not designed for retinal imaging; thin-vessel continuity difficult
Fu et al. [133]	M-Net (joint network with polar transform)	ORIGA, SCES	Joint optic disc and cup segmentation	State-of-the-art OD/OC segmentation	ORIGA/SCES not public; limited cross-dataset validation
Oktay et al. [131]	Attention U-Net	CT datasets; adapted to retinal vessels	Vessel and organ boundary segmentation	Attention gates consistently improved accuracy	Original validation on CT, not retinal images
Wan et al. [134]	EAD-Net (encoder–attention–decoder)	e-ophtha, IDRiD	DR lesion segmentation	~10% sensitivity and F1 improvement over U-Net	Varies across lesion types; rare lesions are hard to detect
Chen et al. [135]	TransUNet (CNN-Transformer hybrid)	Synapse CT, ACDC MRI; adapted to retina	General medical image segmentation	Outperformed pure CNN and pure transformer baselines	Non-eye-disease benchmarks; higher computational cost
Li & Tao [136]	V-MNet (shared encoder + vessel-guided classification)	RFMiD, ODIR-5K, DRIVE, EyePACS-light-v2	Vessel segmentation + multi-disease classification	Dice 0.831, AUC 0.985; mean AUC 0.978, F1 0.935	Retrospective public datasets; no prospective validation
Oladele et al. [113]	DA-VesselNet, ResNet50 + attention U-Net with domain adaptation	DRIVE, CHASE_DB1, FIVES; target: RFMiD; external validation on STARE	Domain-adaptive retinal vessel segmentation for unannotated fundus images	Improved vessel segmentation across source domains; CHASE_DB1 Dice 0.6097, AUC 0.9569; FIVES Dice 0.7201, AUC 0.9684	RFMiD lacks vessel masks for direct target-domain segmentation evaluation; performance remains dependent on source-domain quality and domain shift

**Table 5:** Clinical-readiness checklist for AI-based disease detection systems.

Readiness Item	Minimum Evidence or Consideration
External validation	Testing on data from sites, devices, and populations not used in development
Prospective evaluation	Assessment in live or near-live clinical workflow rather than only retrospective datasets
Calibration	Probability estimates tested with calibration curves, Brier score, or expected calibration error
Threshold justification	Disease-specific thresholds linked to referral risk, missed-disease cost, and clinical workload
Uncertainty handling	Clear pathway for low-confidence or low-quality images to be reviewed by clinicians
Explainability	Clinician-readable visual or feature-level explanations checked for clinical plausibility
Resource profile	Inference time, model size, hardware needs, and energy use reported for target settings
Regulation clearance	Status and scope of regulatory clearance (e.g., FDA, CE, SAHPRA) and the exact approved intended use
Workflow integration	Fit with existing clinical workflow, referral pathways, and electronic health records
Human factors	Usability for operators and clinicians, and clarity of how model outputs are acted upon
Health economics	Evidence on cost-effectiveness and resource use for the intended deployment setting
Post-market surveillance	Plan for ongoing monitoring of real-world performance after deployment
Data drift monitoring	Detection of shifts in input data distribution or performance over time
Cybersecurity and data governance	Protection of patient data, model integrity, and audit trails
Liability and accountability	Clear allocation of responsibility for AI-assisted clinical decisions
Equity and fairness	Performance audited across demographic, geographic, and imaging-device subgroups

## Data Availability

No new data were created or analyzed in this study.

## Abbreviations

The following abbreviations are used in this manuscript:

AMD	Age-related Macular Degeneration
ANN	Artificial Neural Network
AUC	Area Under the Receiving Operating Characteristic Curve
CBAM	Convolutional Block Attention Module
CDR	Cup-to-Disc Ratio
CNN	Convolutional Neural Network
CNV	Choroidal Neovascularization
DME	Diabetic Macular Oedema
DR	Diabetic Retinopathy
DT	Decision Tree
ICDR	International Clinical Diabetic Retinopathy
KNN	K-Nearest Neighbor
LIME	Local Interpretable Model-agnostic Explanations
ML	Machine Learning
NPDR	Non-proliferative Diabetic Retinopathy
OCT	Optical Coherence Tomography
PCA	Principal Component Analysis
ROP	Retinopathy of Prematurity
SHAP	SHapley Additive ExPlanations
SVM	Support Vector Machine
ViT	Vision Transformer
XAI	Explainable Artificial Intelligence
